# Neuroprotection of dopamine neurons by xenon against low-level excitotoxic insults is not reproduced by other noble gases

**DOI:** 10.1007/s00702-019-02112-x

**Published:** 2019-12-05

**Authors:** Déborah Le Nogue, Jérémie Lavaur, Aude Milet, Juan Fernando Ramirez-Gil, Ira Katz, Marc Lemaire, Géraldine Farjot, Etienne C. Hirsch, Patrick Pierre Michel

**Affiliations:** 1 Sorbonne Université, Institut du Cerveau et de la Moelle Epinière (ICM), Inserm U 1127, CNRS, UMR 7225, Paris, France; 2 Air Liquide Santé International, Campus Innovation Paris, Jouy-en-Josas, France

**Keywords:** Dopamine neurons, Excitotoxicity, Neurodegeneration, Noble gases, Parkinson disease, Xenon

## Abstract

Using midbrain cultures, we previously demonstrated that the noble gas xenon is robustly protective for dopamine (DA) neurons exposed to l-*trans*-pyrrolidine-2,4-dicarboxylate (PDC), an inhibitor of glutamate uptake used to generate sustained, low-level excitotoxic insults. DA cell rescue was observed in conditions where the control atmosphere for cell culture was substituted with a gas mix, comprising the same amount of oxygen (20%) and carbon dioxide (5%) but 75% of xenon instead of nitrogen. In the present study, we first aimed to determine whether DA cell rescue against PDC remains detectable when concentrations of xenon are progressively reduced in the cell culture atmosphere. Besides, we also sought to compare the effect of xenon to that of other noble gases, including helium, neon and krypton. Our results show that the protective effect of xenon for DA neurons was concentration-dependent with an IC_50_ estimated at about 44%. We also established that none of the other noble gases tested in this study protected DA neurons from PDC-mediated insults. Xenon’s effectiveness was most probably due to its unique capacity to block NMDA glutamate receptors. Besides, mathematical modeling of gas diffusion in the culture medium revealed that the concentration reached by xenon at the cell layer level is the highest of all noble gases when neurodegeneration is underway. Altogether, our data suggest that xenon may be of potential therapeutic value in Parkinson disease, a chronic neurodegenerative condition where DA neurons appear vulnerable to slow excitotoxicity.

## Introduction

Noble gases are monatomic gases with very low chemical reactivity, which explains why they are referred as inert gases (Selig et al. 1964; Khriachtchev et al. 2000). Their chemical inertness is due to a filled valence shell that prevents covalent bonds with other molecules under standard temperature and pressure conditions (Spaggiari et al. 2013). Despite that, xenon and also other noble gases exhibit interesting biological properties (Deng et al. 2014; Winkler et al. 2016), probably because of interactions with cell components through noncovalent forces (Liu et al. 2010; Sauguet et al. 2016).

Xenon is without doubt the noble gas whose biological effects are the most robust and best documented. Xenon can operate as an anesthetic (Jordan and Wright 2010; Winkler et al. 2016) and it possesses analgesic properties (Esencan et al. 2013). Xenon also exerts rapid antidepressant-like effects (Dandekar et al. 2018) and ameliorates l-dopa-induced dyskinesia in experimental Parkinsonism (Baufreton et al. 2018). It has also the capacity to reduce lesions after brain ischemic damage (Thoresen et al. 2009; Lobo et al. 2013) and to provide organ protection before transplantation (Zhao et al. 2017).

Some of the other noble gases also exert interesting biological effects. Argon has demonstrated anti-apoptotic, organoprotective and neuroprotective effects in a number of in vitro and in vivo studies (Broad et al. 2016; David et al. 2012; Spaggiari et al. 2013; Zhao et al. 2017). Helium is used as substitute for nitrogen to prevent nitrogen narcosis in diving (Berganza and Zhang 2013). Besides, helium was reported to protect myocardial tissue (Oei et al. 2012) and brain neurons (Pan et al. 2007) in preclinical models of ischemia reperfusion. Krypton and neon have not been described as having significant biological effects until now (Jawad et al. 2009; Spaggiari et al. 2013), even if krypton has been found to induce anesthesia under hyperbaric pressure (Cullen and Gross 1951).

We reported previously that xenon promotes neuronal rescue not only against acute ischemic insults as initially demonstrated, but also in experimental situations where neurodegeneration is more slowly progressing (Lavaur et al. 2016a, b). In particular, xenon was found to protect dopamine (DA) neurons in a culture setting where these neurons undergo sustained, low-level excitotoxicity mediated by glutamate (Lavaur et al. 2017), i.e., experimental conditions that model Parkinson disease (PD) neurodegeneration (Nafia et al. 2008). Indeed, an excessive excitatory drive from subthalamic nucleus glutamatergic neurons is thought to contribute to the vulnerability of substantia nigra DA neurons in this disorder (Wallace et al. 2007; Ambrosi et al. 2014).

Specifically, this slow excitotoxic process was mimicked by treating midbrain dopaminergic cultures with l-*trans*-pyrrolidine-2,4-dicarboxylate (PDC), a synthetic analog of glutamate that operates as a transportable inhibitor of inward glutamate transport (Blitzblau et al. 1996; Grewer et al. 2014). In such paradigm, DA neurons were protected when 75% nitrogen contained in the control atmosphere was substituted with 75% xenon (Lavaur et al. 2017) but we did not explore the neuroprotective potential of lower concentrations of xenon in this setting. Furthermore, even if we reported previously that argon was inactive against DA cell death induced by PDC, we have no information about the effects of other noble gases in the same experimental context. Our two present aims were therefore (i) to determine whether DA cell rescue remains observable when concentrations of xenon are progressively reduced in the cell culture atmosphere and (ii) to study whether helium, neon or krypton can reproduce xenon’s neuroprotective effects.

## Materials and methods

### Pharmacological reagents and pure gases

The synthetic analog of glutamate, PDC (#0298/10) and the blocker of *N*-methyl-d-aspartate (NMDA) receptors, memantine (#0773/50) were both from Tocris Biotechne (Lille, France). Compressed gas cylinders of individual pure gases were provided by Air Liquide (France).

### Midbrain cell cultures

Animal care and experiments were conducted in accordance with recommendations of the European Union Council Directives (2010/63/EU). Experimental procedures for cell cultures were authorized under No. 0037 by the ethical committee on animal experiments Charles Darwin No. 5.

Cultures were prepared using gestational day 15.5 embryos from Wistar female rats (Janvier LABS, Le Genest St Isle, France) that had been deeply anesthetized with sodium pentobarbital and killed by cervical dislocation. Once dissected, ventral midbrains were processed to obtain dissociated midbrain cultures following procedures described previously in detail (Toulorge et al. 2011; Lavaur et al. 2017). Midbrain cells were then plated at a density of 1.2–1.5 × 10^5^ cells/cm^2^ onto Nunc 48 well multidish plates (ThermoFisher Scientific, Roskilde, Denmark) pre-coated with 1 mg/ml polyethylenimine diluted in borate buffer, pH 8.3 as described (Sepulveda-Diaz et al. 2016). These cultures which contained about 2–3% of tyrosine hydroxylase (TH) positive neurons, at the time of plating, were maintained in Neurobasal medium (Gibco, Saint Aubin, France) supplemented with a B27 cocktail minus antioxidants (Gibco), a N_2_ mix (Gibco), 2 mM glutamine and 100 IU/ml penicillin/streptomycin (Nafia et al. 2008; Lavaur et al. 2017).

### Treatment procedures

#### Pharmacological treatments

Treatments with the NMDA receptor blocker memantine and the inhibitor of glutamate transport PDC were initiated just before starting the incubations with the gaseous atmospheres at 12 days in vitro (DIV) and terminated at 16 DIV. The culture medium was never changed during the entire culture time. These conditions were previously reported to allow for optimal development and maintenance of DA neurons in control culture conditions (Lavaur et al. 2017).

#### Exposure to gas atmospheres

After application of pharmacological treatments, culture plates were positioned within plexiglas incubation chambers (CR1601, EnzyScreen, The Netherlands) filled with humidified gas combinations just above atmospheric pressure. Specific gas concentrations were produced with a gas mixing system (GasMix, Alytech, Juvisy/Orge, France) supplied by compressed gas cylinders of individual pure gases. The description of the technical procedure used to fill up incubation chambers with gas atmospheres has been previously described in detail (Lavaur et al. 2016a, 2017). The control cell culture atmosphere contained 20% O_2_, 5% CO_2_ and 75% N_2_ and test atmospheres adequate percentages of noble gases in replacement of an equivalent amount of nitrogen. The incubation chambers were kept at 37 °C within conventional cell culture incubators for the duration of the treatments. When using helium, the gas atmosphere was replenished daily and silicone grease was applied to renew the gas tight seal of the incubation chamber because helium has a greater tendency to leak.

### Detection of dopamine neurons by immunofluorescence

After fixation with 4% formaldehyde in Dulbecco’s phosphate buffered saline (PBS), midbrain cultures were washed twice with PBS before an incubation step at 4 °C for 96 h with a rabbit polyclonal anti-TH antibody (T-9237-13; US Biologicals, Salem, MA, USA) diluted 1:1000 in PBS containing 0.2% Triton X-100 to favor cell membrane permeability. The primary antibody was detected with an Alexa Fluor-555 conjugate of an anti-rabbit antibody (1:1000) obtained from Life Technologies (Saint Aubin, France). All TH^+^ neurons detected in this type of cultures were reported to have a dopaminergic phenotype (Traver et al. 2006). Cultures were counterstained with 4′,6-diamidino-2-phenylindole (DAPI) to enable automated focus during the cell counting procedure.

### Counting of TH^+^ neurons

Individual digitized images of DAPI^+^ nuclei (blue) and TH^+^ cells (red) were acquired under a square format using an Arrayscan XTi automated fluorescence microscope fitted with a 10× objective (ThermoScientific, Courtaboeuf, France). After acquisition of about 64% of the total surface area of each culture well (i.e., the square inscribed in the perimeter of the culture well), a merged composite image was generated for quantification of TH^+^ neurons with the HCStudio software (ThermoScientific, Courtaboeuf, France). Only TH^+^ cell bodies with undamaged DAPI^+^ nuclei and at least one neuritic extension were counted as surviving DA neurons.

### Mathematical modeling of gas diffusion in culture wells

We also performed calculations to estimate theoretical concentrations of noble gases achievable in culture wells, at the level of cell layers. Precisely, a concentration of 50% was chosen for modeling one-dimensional gas diffusion and the depth of the liquid column corresponding to 500 µl of culture medium in culture wells was estimated to be 6 mm.

The mathematical model and experimental parameters used to perform calculations have been described in detail in one of our previous papers (Katz et al. 2016). In brief, the transport of the Noble gas through the liquid medium to the cells is modeled as a one-dimensional diffusion problem:

1 $$ \frac{\partial C}{\partial t} = D\frac{{\partial^{2} C}}{{\partial y^{2} }}, $$

where *C* is the concentration in the liquid and *D* is the diffusion coefficient in the liquid medium (we assume the *D* for water). The boundary conditions are no flux at the bottom of the well at *y *= *0* and saturated at the surface at *y *= *L*:

2 $$ \frac{{\partial C\left( {0,t} \right)}}{\partial y} = 0,\quad C\left( {L,t} \right) = C_{\text{sat}} . $$

The initial condition is: $C\left( {y,0} \right) = 0$ except at the water surface *y *= *L*, where it is assumed to be saturated, *C *= *C*_sat_. The solution to this differential equation in the form of a Fourier series is given by

3 $$ C\left( {y,t} \right) = C_{\text{sat}} - C_{\text{sat}} \sum\nolimits_{n = 0}^{\infty } {\left[ {\frac{{2\;\left( { - 1} \right)^{n} }}{{\left( {n + {\raise0.7ex\hbox{$1$} \!\mathord{\left/ {\vphantom {1 2}}\right.\kern-0pt} \!\lower0.7ex\hbox{$2$}}} \right)\pi }}e^{{ - \left( {n\; + \;{\raise0.7ex\hbox{$1$} \!\mathord{\left/ {\vphantom {1 2}}\right.\kern-0pt} \!\lower0.7ex\hbox{$2$}}} \right)^{2} \pi^{3} {\text{Fo}}}} \cos \left( {\left( {n + \frac{1}{2}} \right)\pi \frac{y}{L}} \right)} \right]} , $$

where Fo is the Fourier number for nondimensional time: Fo= *Dt/L*^*2*^.

For calculations, we used diffusion and solubility constants of noble gases in water, at 37 °C (Table 1). Precisely, diffusion data were from Langø et al. (1996) and Nepal and Adhikari (2017). Solubility coefficients were from Wilhelm et al. (1977) and we used interpolated values between 35 and 40 °C. We also chose to provide estimated values, at 2 h of PDC treatment when neurodegeneration is not truly engaged and at 8 h when first neurodegenerative changes are visually observed.

**Table 1:** Diffusion and Ostwald solubility coefficients of noble gases in water, at 37 °C

Noble gas	Diffusion coefficient (× 10^−5^ cm^2^/s)	Ostwald solubility coefficient
Xe	1.55	0.083374
Kr	2.0	0.050364
Ar	2.9	0.02964
Ne	4.8	0.010922
He	10	0.009781

Diffusion (10^−5^ cm^2^/s) and Ostwald solubility coefficients used for mathematical modeling of noble gas diffusion in water, at 37 °C. Diffusion data were from Langø et al. (1996) and Nepal and Adhikari (2017). Solubility coefficients were from Wilhelm et al. (1977) and we used interpolated values between 35 and 40 °C

*Xe* Xenon, *Kr* krypton, *Ar* argon, *Ne* neon, *He* helium

### Statistical analysis

Data expressed as mean ± SEM were analyzed using the SigmaPlot 12.5 software (Systat Software Inc, San Jose, CA, USA). Statistical analysis was performed with a one-way ANOVA followed by a Student–Newman–Keuls post hoc test to perform all pairwise comparisons. Estimation of the IC_50_ value was made with a four-parameter logistic regression model.

## Results

**Fig. 1:**
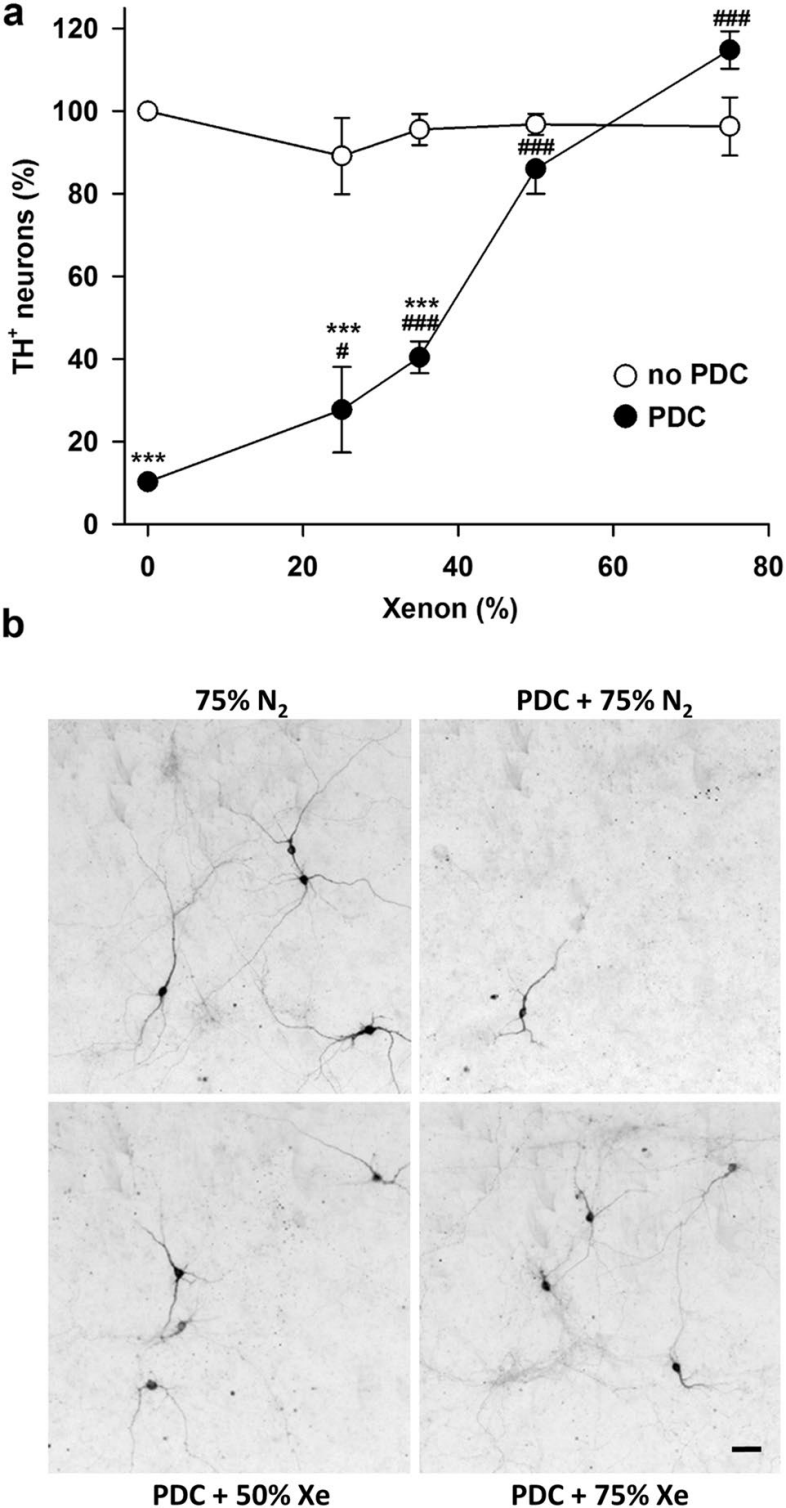
Xenon provides concentration-dependent protection against PDC-induced DA cell death in midbrain cultures. **a** Survival rate of DA neurons (TH^+^ cells) in 16 DIV midbrain cultures previously exposed or not for 4 consecutive days to PDC (100 µM) under cell culture atmospheres containing 75% N_2_ or 25–75% Xe. Error bars indicate mean ± SEM (*n* = 2–8). ***p < 0.001 relative to control cultures maintained under 75% N_2_. #*p* < 0.05 and ^###^*p* < 0.001 relative to PDC-treated cultures maintained under 75% N_2_. Application of a 4-parameter logistic regression model to experimental data values gave an IC_50_ of 44.4% for Xe. **b** Inverted fluorescence images illustrating the neuroprotective effects provided by 50% and 75% Xe in midbrain cultures exposed for 4 days to 100 µM of PDC. Scale bar 55 µm

We used the synthetic analog of glutamate PDC to generate, in vitro, low-level excitotoxicity as it may occur in PD (Wallace et al. 2007; Ambrosi et al. 2014; Michel et al. 2016). More precisely, midbrain cultures maintained initially for 12 DIV in serum-free conditions, were then exposed for 4 consecutive days to 100 µM PDC to induce the death of DA (TH^+^) neurons as described previously (Lavaur et al. 2017). In PDC-treated cultures maintained under a control atmosphere consisting of 75% nitrogen, 20% O_2_ and 5% CO_2_, the survival rate of TH^+^ neurons was decreased by about 90% at 16 DIV (Fig. 1a). When nitrogen was substituted with 75% xenon, DA cell death was virtually absent from the cultures (Fig. 1a, b). Of interest, the survival of TH^+^ neurons was also significantly improved under an atmosphere where the concentration of xenon was set at 50%. The survival rate of TH^+^ neurons in these conditions was 86% of that of control (PDC-free) cultures maintained under 75% nitrogen (Fig. 1a, b). The rescuing effect of xenon was progressively reduced at lower concentrations but remained significant with 35% and 25% of the noble gas in the cell culture atmosphere. This set of data allowed us to estimate at 44.4% the concentration of xenon reducing by half (IC_50_) the death of DA neurons. Note that we also quantified the survival rate of TH^+^ neurons in PDC-free cultures exposed to various concentrations of xenon (25–75%). There was no significant change in TH^+^ cell numbers in these conditions, regardless of the concentration of xenon applied to the cultures (Fig. 1a).

**Fig. 2:**
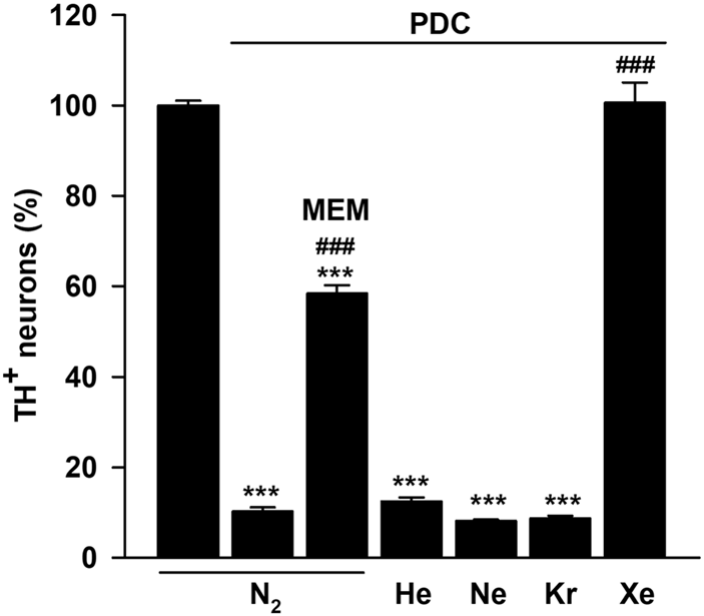
Among noble gases, only xenon protects DA neurons against PDC-induced degeneration in midbrain cultures. Survival rate of TH^+^ cells in midbrain cultures exposed to PDC (100 µM) for 4 days under a control culture atmosphere containing 75% N_2_ or other atmospheres enriched with 75% of He, Ne, Kr or Xe. Comparison with a non-gaseous treatment by memantine (MEM) (10 µM) performed under 75% N_2_. Error bars indicate mean ± SEM (*n* = 3–8). ***p < 0.001 relative to control cultures maintained under 75% N_2_. ^###^*p* < 0.001 relative to PDC-treated cultures maintained under 75% N_2_

We then compared the effect of xenon to that of other noble gases, including helium, neon and krypton. More specifically, we estimated the survival of TH^+^ neurons in PDC-treated cultures maintained for 4 days under gas atmospheres where nitrogen was substituted with 75% of each of these gases. Xenon was used as reference gas in this context. As shown previously, a treatment of midbrain cultures with 100 µM PDC caused a profound loss of TH^+^ neurons in cultures maintained under a control atmosphere containing 75% nitrogen. As expected, this loss was prevented when nitrogen was substituted with 75% xenon but the other atmospheres containing 75% of helium, neon or krypton in their composition were totally ineffective (Fig. 2). Note that the blocker of NMDA receptors, memantine (10 µM) used as a non-gaseous reference neuroprotective treatment for DA neurons, provided robust but partial protection against PDC under 75% nitrogen (Fig. 2).

Finally, a theoretical estimation made with gaseous atmospheres containing noble gases at 50% in their composition, revealed that xenon was the slowest to saturate the culture medium at the level of the cell layer (Table 2). Xenon together with krypton yielded, however, the highest concentrations of all noble gases, after 2 h of PDC treatment (Table 2). Of interest, the concentration of xenon was well above that of any of the other noble gases including krypton after 8 h of PDC exposure, i.e., at a stage where first neurodegenerative changes are visually observable in our culture set-up. In particular, a number of swollen neuronal cell bodies were detectable at this time-point by phase-contrast microscopic examination of living cultures. Note that helium was the noble gas yielding the lowest concentrations after 2 and 8 h of PDC exposure. Argon and neon generated concentrations that were intermediary at both time points.

**Table 2:** Mathematical estimation of noble gas concentrations in culture wells, at the cell layer level

Noble gas	Concentration (mmol/L)		Medium saturation (%)	
	2 h	8 h	2 h	8 h
Xe	0.668	1.637	40.8	94.2
Kr	0.685	0.989	69.3	99.5
Ar	0.407	0.585	69.6	99.6
Ne	0.207	0.216	95.8	100
He	0.193	0.193	99.9	100

Mathematical modeling of gas diffusion in the culture medium for an estimation of gas concentrations at the cell layer level (depth of 6 mm of the liquid column). Theoretical estimations are given after 2 and 8 h of incubation with gaseous atmospheres containing noble gases at a concentration of 50%. Saturation rates of medium are shown at corresponding time points

## Discussion

The present work allowed us to further characterize the protective effects of xenon for midbrain DA neurons in culture and to compare its effects to that of other noble gases in the same experimental setting. Using the synthetic analog of glutamate PDC to generate sustained low-level excitotoxicity as it may occur in PD (Wallace et al. 2007; Assous et al. 2014), we confirmed the strong protective potential of xenon for DA neurons. Importantly, we also showed that the effects of xenon were concentration-dependent with optimal effects observed at concentrations comprised between 50 and 75%. Note that we noticed that the atmosphere containing 75% xenon was comparatively more effective in protecting DA neurons in the present study than in a research work we conducted earlier with the same culture system (Lavaur et al. 2017). The experimental set-up and culture conditions being identical in these two studies, we have no clear explanation for such a difference.

By performing non-linear regression analysis of current data values, we estimated at 44.4%, the IC_50_ for neuroprotection by xenon. The IC_50_ established by us appears higher than IC_50_ values reported previously for xenon-mediated neuroprotection (Wilhelm et al. 2002). More specifically, in cortical cultures submitted to acute exposures to NMDA and glutamate, IC_50_ values for xenon were estimated at 19% and 28%, respectively. Such a difference may relate to the fact that we induced excitotoxic stress by means of a treatment with PDC, a toxin that does not act as a direct agonist at glutamate receptors but operates indirectly by causing a small but permanent rise of extracellular glutamate (Maki et al. 1994; Lavaur et al. 2017). Furthermore, a prolonged exposure to PDC for 4 days might have made xenon less efficacious during the course of treatment. This explanation appears, however, pertinent only when considering the lowest concentrations of xenon (i.e., 25% and 35%) and their limited efficacy to rescue DA neurons from PDC exposure in our paradigm.

At concentrations of 50% or above, xenon was reported to be highly effective in reducing NMDA receptor-mediated currents (Dickinson et al. 2007; Haseneder et al. 2009), which probably explains why this noble gas did provide robust protection under these conditions. Consistent with this view, DA cell death induced by PDC was also efficiently reduced by antagonizing NMDA receptors with memantine, in cultures maintained under 75% nitrogen. Note that the site to which xenon binds on NMDA receptors appears distinct from that of memantine (Kotermanski and Johnson 2009; Liu et al. 2010) and unrelated to the glycine binding site of the receptor in the present setting (Lavaur et al. 2016a). Indeed, glycine is inherently present in Neurobasal culture medium at a concentration of 400 μM, which is sufficient to saturate the NMDA receptor glycine-binding site (Dickinson et al. 2007).

We also found that in cultures not treated with PDC, TH^+^ cell numbers were unaffected by xenon regardless of the gas concentration used for treatment. This result indirectly demonstrates the lack of toxicity of xenon in present culture conditions. It also shows that xenon did not mimic the effects of some synthetic compounds and biological molecules reported to induce the expression of the TH enzyme in dormant neurons not initially committed to this neurotransmitter phenotype (Stull et al. 2001; Traver et al. 2006). This indicates that xenon operated as a true neuroprotectant in the present paradigm.

We also tested whether other noble gases, including helium, neon and krypton had the potential to rescue DA neurons in the same experimental paradigm. Based on the literature, the rational for testing neon and krypton is relatively limited. Helium, however, has been reported to provide protection to brain neurons in preclinical models of ischemia reperfusion (Pan et al. 2007) and traumatic brain injury (Coburn et al. 2008). Our results show that neither helium, neon nor krypton had the capacity to protect PDC-treated DA neurons from degeneration when the concentration of these gases in the cell culture atmosphere was set at 75%. Note that despite a number of studies reporting on the protective effects of argon in preclinical models of acute ischemic/anoxic stress (Coburn et al. 2012; Zhao et al. 2017), we have previously established that argon was also ineffective to protect DA neurons from PDC-induced degeneration (Lavaur et al. 2017). This suggests that among noble gases, only xenon has the potential to provide protection when neurodegeneration is sustained and slowly progressing.

One may assume that this property is related to the fact that xenon possesses the highest atomic number and therefore the largest atomic radius of all noble gases (The International Union of Pure and Applied Chemistry (IUPAC) 2018; Zhang and Xu 1995), a feature that may be correlated to the capacity of xenon to block NMDA receptors. Coherent with this view, argon, krypton, neon and helium, all failed to interfere with NMDA receptor activity (Harris et al. 2013).

Note that we cannot exclude, however, that favorable PK properties also contributed to the efficacy of xenon. In particular, in this in vitro experimental set-up, the concentrations of xenon at the level of the cell layer and the time necessary to reach these concentrations may be important factors for neuroprotection (Katz et al. 2016). Theoretical calculations made with gaseous atmospheres containing noble gases at a concentration of 50%, revealed that xenon was the slowest of all these gases to saturate the culture medium. Despite of that, xenon yielded the highest theoretical concentrations in the culture medium, at the level of the cell layer. This was particularly true after 8 h of PDC exposure, i.e., when first neurodegenerative changes are observable in the cultures. Therefore, our data suggest that xenon protective effects may relate not only to specific pharmacological properties but also to its availability for neuronal cells. However, it is clear that these in vitro PK results do not consider the clinically relevant physiological transport to and location of the cellular targets in vivo (e.g., blood flow to the brain, partition coefficient and lipophilicity of the gas molecule…).

Overall, we have demonstrated that xenon is particularly effective in the present experimental setting that models the slow excitotoxic component of DA cell death in PD. It remains to be demonstrated, however, whether such properties are clinically relevant.
